# ADAM17-mediated CD16 shedding drives monocyte subset misclassification in ex vivo immunological assays

**DOI:** 10.3389/fimmu.2026.1886931

**Published:** 2026-10-13

**Authors:** Soyeon Kwon, Seung Kak Shin, Seungwon Ryu

**Affiliations:** 1 Department of Bio-Medical Science, GAIHST, Gachon University, Incheon, Republic of Korea; 2 Department of Microbiology, Gachon University College of Medicine, Incheon, Republic of Korea; 3 Division of Gastroenterology and Hepatology, Department of Internal Medicine, Gachon University Gil Medical Center, Gachon University College of Medicine, Incheon, Republic of Korea

**Keywords:** ADAM17, CD16 shedding, flow cytometry, intracellular cytokine staining, monocyte

## Abstract

Human monocytes are classified as classical (CD14++CD16−), intermediate (CD14++CD16+), and non-classical (CD14+CD16++) subsets, but CD16 stability during ex vivo assays remains uncertain. Here, we examined whether standard PBMC handling alters CD16-based subset identity and whether ADAM17 inhibition can prevent this artifact. In isolated PBMCs, intermediate and non-classical monocytes rapidly lost surface CD16 during culture at 37 °C, with or without LPS stimulation, whereas CD16 expression remained stable at 4 °C and in unseparated whole blood. Pre- versus post-incubation labeling and sorted-cell experiments indicated that CD16 loss was not primarily due to receptor internalization or cell death, but instead produced a pseudo-classical phenotype. Public scRNA-seq analyses showed enriched ADAM17 expression in monocyte/macrophage populations across blood, liver, and kidney datasets. Pharmacological ADAM17 inhibition with TMI-005 or TAPI-1 preserved CD16 expression during LPS stimulation without broadly impairing monocyte cytokine production. We therefore propose an ADAM17 inhibitor-based protocol that combines surface staining, stimulation, and intracellular cytokine profiling to preserve monocyte subset identity during functional assays. These findings identify a practical strategy to reduce CD16-dependent monocyte subset misclassification in *ex vivo* immunological studies.

## Introduction

Monocytes are a heterogeneous population of immune cells that have been classified into three major subsets on the basis of their surface expression of CD14 (a co-receptor for Toll-like receptor-4) and CD16 (Fcγ receptor-III): classical monocytes (CD14++ CD16−), intermediate monocytes (CD14++ CD16+), and non-classical monocytes (CD14+ CD16++) (1, 2). Classical monocytes constitute the majority (approximately 85-90%) of circulating monocytes and are characterized by their pronounced recruitment to sites of injury and inflammation (3). Intermediate monocytes are much less abundant than classical monocytes and express higher levels of HLA-DR and Toll-like receptors. They also demonstrate more potent antigen-presenting capacity and cytokine production on stimulation and are thus considered a pro-inflammatory monocyte subset (4, 5). Non-classical monocytes also constitute a small subset of monocytes, and their most well-known function is to patrol endothelial surfaces, thereby contributing to immune surveillance (6–8). Thus, each subset has distinct functional roles and phenotypes.

Multiple studies indicate that classical monocytes convert into intermediate monocytes, while intermediate monocytes differentiate into non-classical monocytes (9, 10). This plasticity, which alters monocyte expression of CD14 and CD16, may be rapidly triggered by environmental stimuli such as inflammation, infection, and stress (11, 12). This likely explains why the relative proportions of the three monocyte subsets in the circulation can change in various pathological conditions. For example, chronic inflammatory diseases such as rheumatoid arthritis (13) and sepsis (14) associate with significant expansion of intermediate monocytes. Interestingly, however, acute diseases often associate with apparent loss of the CD16+ intermediate and non-classical subsets from the circulation (10). While it is possible that this reflects CD16+ monocyte death or recruitment out of the circulation, another possibility is that the monocytes shed CD16. This is supported by the well-known fact that natural killer (NK) cells are regulated by CD16 proteolysis by ADAM17, a cell-membrane metalloproteinase: the consequent shedding of CD16 prevents NK-cell overstimulation (15, 16). Significantly, a few studies have reported that monocytes also undergo CD16 loss after in vitro culture in inflammatory conditions (17, 18). Moreover, one of these studies showed that this CD16 loss could be reversed by an ADAM17 inhibitor (18). Thus, acute inflammation and possibly also simple ex vivo culture could lead intermediate and non-classical monocytes to shed CD16 via ADAM17, thus causing them to artifactually resemble classical monocytes. However, this phenomenon has not been examined in detail.

These observations have led to questions about the reliability of CD16 as a definitive lineage marker of monocytes. This doubt has also been significantly compounded by findings that simply culturing monocytes may also induce CD16 loss (17, 18). This is a serious methodological limitation that has likely often affected data interpretation, because cells that have lost CD16 will be misidentified or excluded from subset-specific flow-cytometric analyses. Nonetheless, CD16 is a classical marker of monocyte classification, and alternative monocyte classification systems that do not rely on CD16 (17–19) are limited by their complexity, cost, and limited uptake by the research community. Moreover, although several additional surface markers have been proposed to define monocyte subsets, their stability and robustness across diverse inflammatory and stimulatory conditions have not been systematically validated, and it remains unclear whether any of these markers provides greater reliability than CD16.

While Waller et al. first demonstrated that ADAM17-mediated shedding contributes to the apparent loss of CD16+ monocytes under specific inflammatory stimulation conditions (18), several critical aspects of this phenomenon remain uncharacterized. First, it remains unclear whether simple ex vivo culture without any inflammatory stimulus is sufficient to drive CD16 shedding, and if so, under what temperature and time conditions this occurs. Second, the possibility that CD16 loss reflects receptor internalization rather than proteolytic cleavage has not been directly excluded by experimental evidence. Third, whether the context of whole blood versus isolated PBMCs differentially affects CD16 stability has not been systematically examined. Fourth, the expression of ADAM17 across monocyte subsets and across multiple tissue-resident immune populations has not been comprehensively characterized. Finally, and most importantly from a practical standpoint, a validated experimental protocol that integrates ADAM17 inhibition into simultaneous phenotypic and functional analysis of monocyte subsets — including intracellular cytokine staining — has not been established. Addressing these gaps is essential for developing reliable experimental standards for human monocyte research.

Thus, to improve monocyte research, we here carefully delineated the effect of culture with and without inflammatory stimuli on the CD16 expression of monocytes. We also comprehensively characterized ADAM17 expression in the monocyte subsets. Notably, we found that pharmacologically inhibiting ADAM17 effectively prevented CD16 shedding during inflammatory stimulation. This finding led us to develop an experimental protocol that is based on ADAM17 inhibition: we show here that this protocol prevents CD16 loss, therefore preserving the true functional identity of monocyte subsets during prolonged ex vivo manipulation.

## Materials and methods

### Human samples and cell preparation

Human blood samples from adult volunteers were obtained with informed consent under protocols approved by the Institutional Review Board of Gachon University Gil Medical Center (Incheon, South Korea) (Approval No. GAIRB2023-241). Blood was processed as follows. Peripheral blood mononuclear cells (PBMCs) were isolated by density-gradient centrifugation (Ficoll-Paque Plus; GE Healthcare, Cat# 17-1440-02) according to standard protocols (20), washed, and resuspended in complete RPMI 1640 medium, namely, RPMI 1640 with L-glutamine (Biowest, Cat# L0500-100) supplemented with 10% fetal bovine serum (Biowest, Cat# S1480) and gentamicin (Gibco, Cat# 15710064). Residual red blood cells were lysed by using ammonium chloride-based RBC Lysis Buffer (Biolegend, Cat# 420301) according to manufacturer instructions. Cell numbers and viability were assessed by the Trypan blue exclusion method with a SOL Count automated cell counter (Sol Inc.). For short-term storage, PBMCs were cryopreserved at -80 °C in freezing medium (Cell Banker 1, Zenoaq) and used within four weeks. Upon thawing, cells were immediately transferred to pre-warmed complete RPMI 1640, washed twice, and kept at 4 °C for no longer than 30 minutes before use in all functional assays. Unless otherwise stated, cryopreserved PBMCs were used for all experiments; freshly isolated PBMCs were used for the comparison of whole blood and isolated PBMCs shown in **Supplementary Figure S1**. All experimental procedures involving human cells were carried out in accordance with institutional guidelines and the Declaration of Helsinki.

### Monocyte stimulation, culture and ADAM17 inhibition

For functional assays, PBMCs were plated at 2–5 × 10^5^ cells per well in 96-well plates (200 µL per well). To prevent CD16 shedding, cells were immediately preincubated for 20 minutes on ice with 1 µg/mL of the ADAM17 inhibitors Apratastat (TMI-005; Sigma-Aldrich, Cat# SML1543) or TAPI-1 (TargetMol, Cat# T6009). Control wells received an equivalent volume of DMSO (0.2% v/v; Sigma-Aldrich, Cat# D2438) as a vehicle control. In a separate set of experiments, cells were preincubated under the same conditions with the ADAM10/ADAM17 inhibitor GW280264X (1 µM; Tocris Bioscience, Cat# 7030) or the ADAM10-preferential inhibitor GI254023X (1 µM; Tocris Bioscience, Cat# 3995), each dissolved in DMSO at the same final concentration. Simultaneously, cells were stimulated with 80 ng/mL lipopolysaccharide (LPS) (from Escherichia coli O111:B4; Sigma-Aldrich, Cat# L4391) for 4–6 hours at 37 °C. Unless otherwise stated, surface staining for CD14 and CD16 was carried out at the same time as stimulation rather than before or after it; the two approaches are compared directly in **Supplementary Figure S4**. This stimulation protocol was optimized to elicit robust monocyte activation and cytokine production while remaining within standard experimental ranges. Parallel cultures without LPS (medium alone) were included as unstimulated controls to assess the baseline effects of the inhibitors. After stimulation, cells were gently resuspended and harvested for flow cytometric analysis.

To evaluate long-term subset stability, additional kinetic experiments were performed. Thus, PBMCs were cultured for up to 12 hours in the absence of ADAM17 inhibitors.

### Flow cytometry, monocyte subset gating, and intracellular cytokine staining

Surface staining was performed to identify monocyte subsets. 2-5×10^5^ cells per condition were transferred to polystyrene tubes and washed with PBS. To exclude dead cells, cells were first stained with the Zombie Aqua Fixable Kit (Biolegend, Cat# 423102) for 5 minutes on ice in the dark. Cells were then washed with staining buffer consisting of PBS supplemented with 2% bovine calf serum (Gibco, Cat# 26170-043). To minimize non-specific binding, cells were incubated with human Fc block (TruStain FcX; BioLegend, Cat# 422302) for 10 minutes at 4 °C. The cells were then stained with fluorescently conjugated antibodies for 20 minutes at 4 °C in the dark. Notably, for the relevant experimental groups, ADAM17 inhibitors were added to the staining cocktail at the indicated concentration to ensure continuous stabilization of CD16 expression. To delineate monocyte subsets, the staining panel included anti-CD45 (PerCP-Cy5.5, clone 2D1; Biolegend), anti-CD14 (APC, clone M5E2; Biolegend), and anti-CD16 (PE, clone 3G8; Biolegend). The cells were then washed and fixed with 2% paraformaldehyde (GeneE Solution, Cat# GSP075500) for 15 minutes at 4 °C in the dark. For experiments requiring intracellular cytokine staining, cells were instead treated with a commercial fixation/permeabilization solution, as described further below.

Monocyte gating strategy (**Figure 1A**): Viable cells were gated as Zombie Aqua-negative. After selecting CD45+ leukocytes, monocytes were gated on the basis of CD14 expression. Within the monocyte gate, subsets were resolved on the CD14 vs. CD16 dot plot. Classical monocytes appear as CD14++ CD16–, intermediate monocytes as CD14++ CD16+, and non-classical monocytes as CD14+ CD16 ++. These gates were established by using unstimulated control and fluorescence-minus-one (FMO) controls.

**Figure 1:**
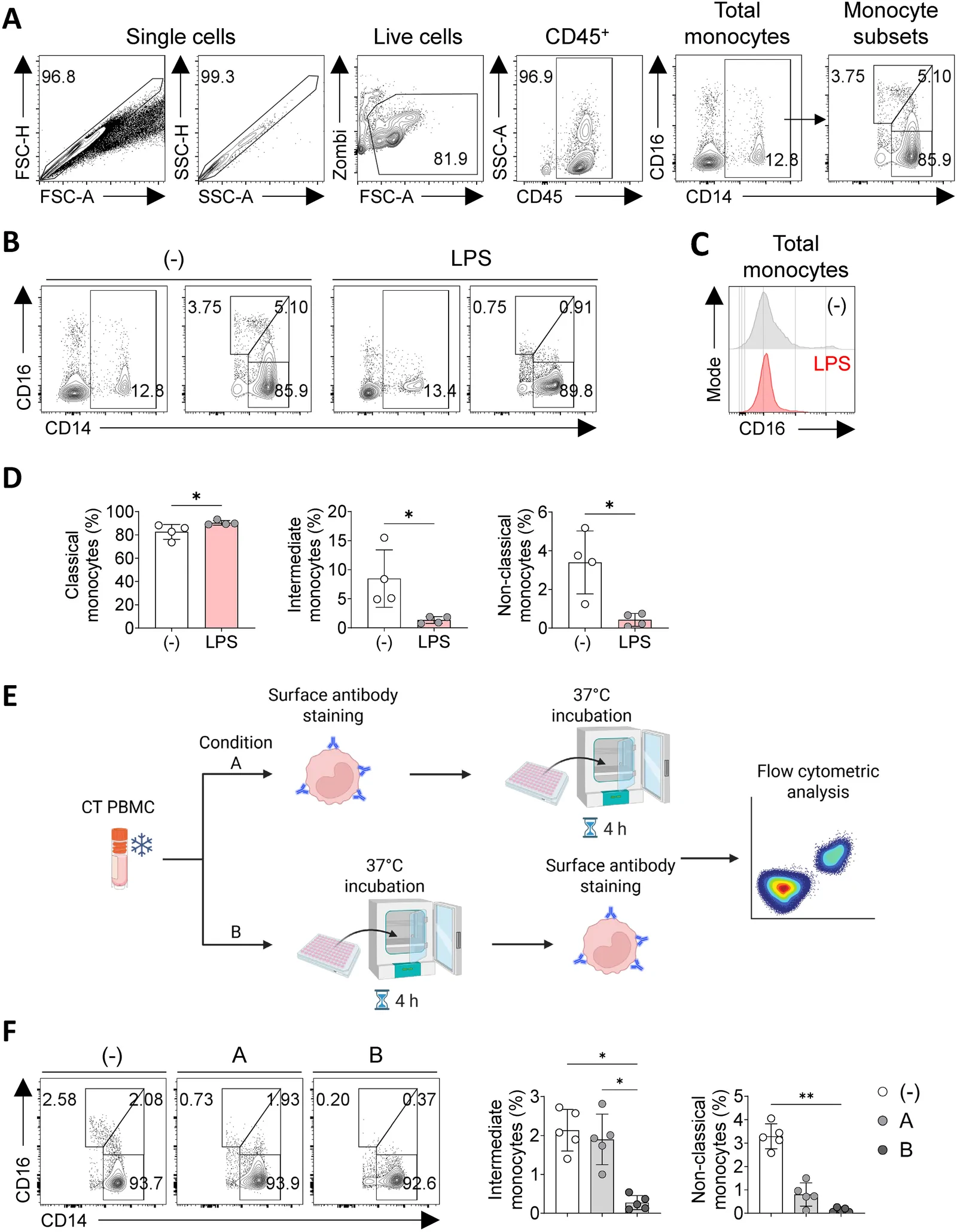
CD16+ monocyte subsets in isolated PBMCs rapidly lose CD16 signals during not only *ex vivo* activation but also culture at 37 °C alone. **(A)** Representative flow-cytometric gating strategy for human monocyte subsets. Total monocytes in human PBMCs were identified from single live CD45+ cells by searching for CD14 signals. The monocytes were then subdivided into classical (CD14++CD16-), intermediate (CD14++CD16+), and non-classical (CD14+CD16++) subsets. **(B, C)** Isolated human PBMCs were treated with LPS (80 ng/mL) for 4 hours. The fresh PBMCs [shown in (-)] and LPS treated PBMCs were then subjected to flow cytometry as described in **(A)**. **(B)** Representative flow-cytometry plots. **(C)** Histogram of CD16 expression in isolated PBMCs treated with LPS for 4 hours. **(D)** Frequencies of monocyte subsets (n = 4). **(E)** Schematic depiction of the experiment assessing the role of CD16 internalization in the loss of CD16+ monocytes from cultured PBMCs. In condition A (upper), representing pre-incubation labeling, human PBMCs were incubated at 4 °C for 20 minutes with antibodies against CD16, incubated at 37 °C for 4 hours, and then subjected to flow cytometry. Condition B (lower) represents post-incubation labeling, PBMCs were first incubated at 37 °C for 4 hours, then labeled with antibodies at 4 °C for 20 minutes and subjected to flow cytometry. **(F)** Comparison of the intermediate and non-classical monocyte frequencies after PBMCs were treated with the A or B labeling conditions depicted in **(E)**. The monocyte-subset frequencies in the fresh PBMCs are indicated by **(-)** (n=5). The data are shown as mean ± SD. *p < 0.05, **p < 0.01, as determined by Mann-Whitney U test or Kruskal-Wallis test with Dunn’s *post-hoc* correction.

Intracellular cytokine staining: For cytokine profiling, we subjected monocytes to intracellular cytokine staining after they were stimulated with LPS. Specifically, during the LPS stimulation, monensin (GolgiStop, BD Biosciences, cat# 554724) was added at 2.0 µM to each well to block cytokine secretion and trap cytokines intracellularly. After washing, the cells were fixed and permeabilized using an intracellular staining buffer set (Cytofix/Cytoperm kit, BD Biosciences, Cat# 554715) and then stained for cytokines. The antibodies used for intracellular staining were anti-TNF-α (Brilliant Violet 785, clone MAb11; Biolegend), anti-IL-6 (PerCP/Cyanine5.5, clone MQ2-13A5; Biolegend), anti-MCP-1 (PE/Cyanine7, clone 5D3-F7; Biolegend), anti-IL-1β (FITC, clone H1b-98; Biolegend), and anti-ADAM17/TACE (PE; R&D Systems, Cat# FAB9301P). Staining was conducted overnight at 4 °C, followed by washing in perm buffer.

All samples were acquired on a BD FACSymphony A5 flow cytometer (BD Biosciences). The data were analyzed using FlowJo v10 (BD Biosciences). Monocytes were analyzed as described above, and cytokine production was quantified as the percentage of cells within each subset that were positive for the cytokine.

### Monocyte subset sorting

To track phenotype changes over time in a controlled manner, we isolated monocyte subsets by fluorescence-activated cell sorting (FACS). PBMCs were stained with the Zombie- Aqua Fixable Kit (Biolegend) and with antibodies to CD14 and CD16 as described above, and sort gates were set on Zombie Aqua-negative cells for CD16+ monocytes (which signals both intermediate and non-classical monocytes) and CD16− monocytes (classical monocytes only). Sorting was performed on a MA900 cell sorter instrument (Sony Biotechnology). The purity of the sorted fractions was >95%. The sorted monocyte subsets were cultured ex vivo in complete medium as described above for unsorted PBMCs. At various time points, cells were harvested and analyzed for CD16 expression by flow cytometry. This approach allowed us to directly observe the loss over time of CD16 from monocytes that had initially been CD16-positive.

### *ADAM17* expression analysis

To investigate the expression profile of ADAM17 and its potential role in CD16 shedding across different tissues, we subjected several publicly available single-cell RNA sequencing (scRNA-seq) datasets to in silico analysis. Specifically, we examined the ADAM17 mRNA levels in various cell types in the peripheral blood (GSE149689) (21), liver (GSE149614) (22), and kidney (GSE189536) (23).

### Statistical analysis

Statistical analyses were performed using Prism 10 software (GraphPad). The data were expressed as mean ± standard deviation. For comparisons between two groups, we applied the Mann–Whitney U test (two-tailed). For comparisons of three or more groups, a non-parametric ANOVA was used, namely, the Kruskal–Wallis test with Dunn’s post-hoc correction for multiple comparisons (for one-way layouts) or Friedman test (for matched/paired data). In cases where two independent variables were considered (e.g. inhibitor treatment and monocyte subset), two-way analysis of variance was performed on rank-transformed data (non-parametric two-way ANOVA approach) to test for interaction effects, followed by appropriate post-hoc tests. P-value < 0.05 was considered statistically significant.

## Results

### Conventional *ex vivo* culture of PBMCs with inflammatory stimuli associates with rapid loss of CD16-expressing monocyte subsets

To establish the impact of standard experimental procedures on monocyte subset frequencies, we isolated human PBMCs and cultured them at the standard 37 °C temperature for 4 hours with LPS (80 ng/mL), and compared them with freshly isolated PBMCs from the same donors. The fresh PBMCs and 4-hour cultured PBMCs were then subjected to a high-resolution flow-cytometry gating strategy to identify the classical (CD14++CD16−), intermediate (CD14++CD16+), and non-classical (CD14+CD16++) monocytes (**Figure 1A**). This showed that compared to the monocyte frequencies in the fresh PBMCs [indicated by (-)], the frequencies of the intermediate and non-classical subsets dropped profoundly by 84–87%. By contrast, there was a mild increase in the CD16– classical monocytes (**Figures 1B–D**). Thus, CD16+ monocytes in PBMCs largely disappear when they are cultured with inflammatory stimuli.

### *Ex vivo* culture of PBMCs alone also associates with rapid loss of CD16-expressing monocyte subsets

To explain the loss of intermediate/non-classical monocytes, we initially hypothesized that the apparent loss of CD16 on ex vivo handling might be due to CD16 internalization or sequestration during incubation at physiological temperatures. To test this, we compared two experimental conditions: (A) pre-incubation CD16 labeling, where PBMCs were treated with surface-marker antibodies for 20 minutes at 4 °C, followed by incubation at 37 °C for 4 hours, and subjected to flow cytometry; and (B) post-incubation CD16 labeling, where the cells were incubated at 37 °C for 4 hours, then labeled with surface-marker antibodies for 20 minutes at 4 °C and subjected to flow cytometry (**Figure 1E**). If the loss of CD16 identity was merely a result of receptor endocytosis, the pre-incubation labeling at 4 °C would still “display” CD16-associated fluorescence in the cells, thereby preserving the subset-specific signals. Thus, loss of CD16 would be observed in only the cells that underwent post-incubation labeling. However, when the monocyte subset frequencies in the two conditions were compared to those in the fresh PBMCs [indicated by (-)], a significant decline in CD16 expression was observed in both: while pre-incubation labeling preserved more intermediate monocytes than post-incubation labeling, the non-classical monocyte population was drastically reduced in both conditions (**Figure 1F**). Thus, the disappearance of the CD16+ subsets on ex vivo culture is largely not due to surface marker internalization; rather, it seems to reflect a sustained phenotypic shift that leads to the apparent loss of these populations.

### Temperature dependency and kinetics of the CD16 loss of monocytes in isolated PBMCs

To systematically define the conditions that provoke the disappearance of CD16+ monocyte subsets during ex vivo culture, we first evaluated the impact of incubation temperature and duration on monocyte subset frequencies in isolated PBMCs. Compared to the monocyte-subset frequencies in fresh PBMCs (time 0), all monocyte-subset frequencies remained stable over 12 hours when the PBMCs were maintained at 4 °C (**Figures 2A, B**). However, at the physiological temperature of 37 °C, we observed a rapid and profound time-dependent reduction in the frequencies of intermediate and non-classical monocytes (**Figures 2A, B**). This decline started within 1 hour of incubation and reached its nadir within 4 hours of the 12-hour observation period (**Figure 2B**). Thus, loss of CD16+ monocyte identity during standard ex vivo culture is a temperature-sensitive process that occurs within 1–4 hours of starting culture.

**Figure 2:**
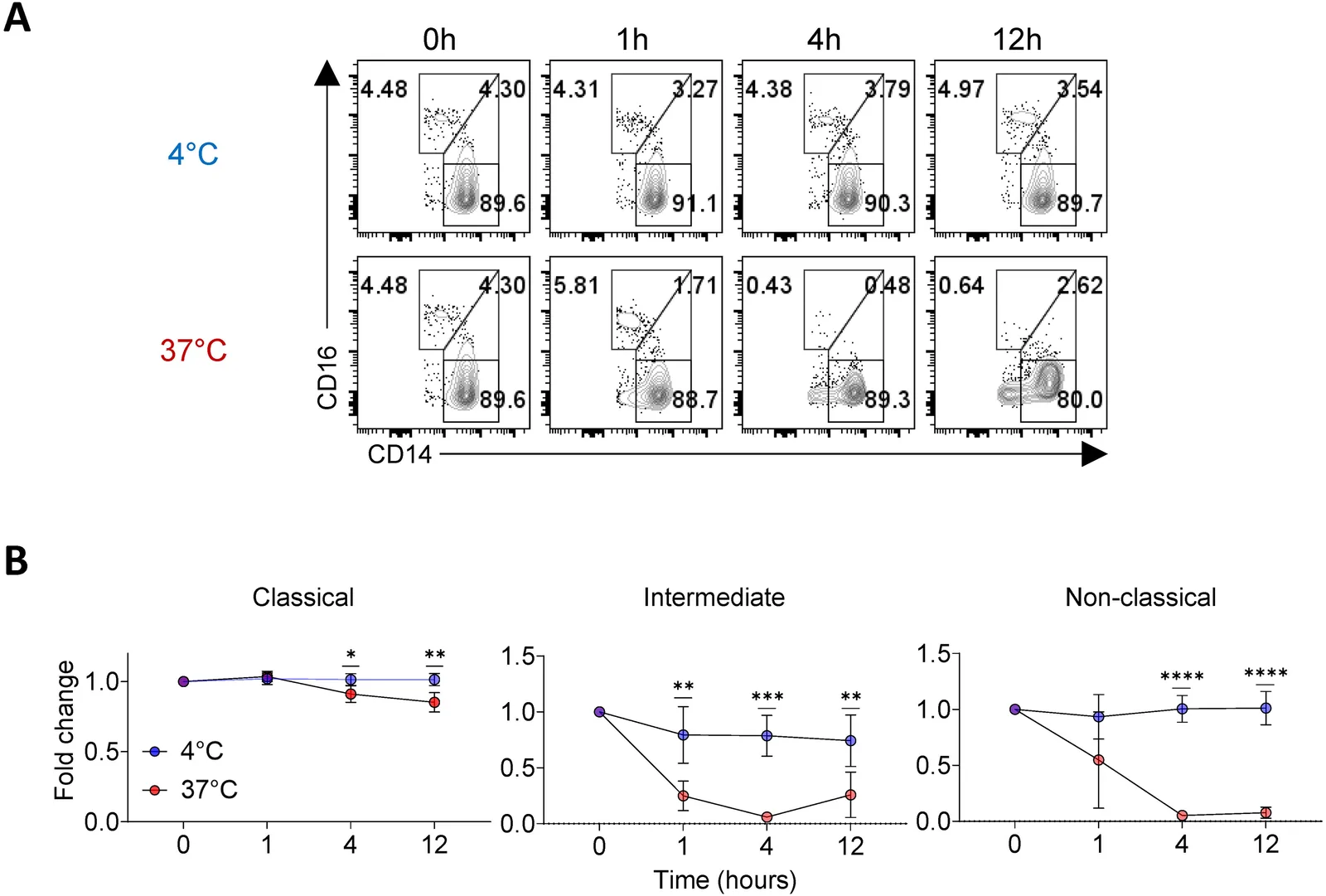
CD16-positive monocyte subsets undergo rapid, temperature-dependent loss in isolated PBMCs. PBMCs were incubated at 4 °C or 37 °C for 0, 1, 4, and 12 hours. **(A)** Representative flow-cytometry plots showing the distribution of monocyte-subset frequencies. **(B)** Fold change in the frequencies of classical (left), intermediate (middle), and non-classical (right) monocyte subsets relative to the fresh PBMCs (0 hour time point). The data are presented as mean ± SD (n = 5). *p < 0.05, **p < 0.01, ***p < 0.001, ****p < 0.0001, as determined by nonparametric two-way ANOVA.

We then asked whether this loss of CD16 expression is restricted to isolated PBMCs or whether it also occurs in whole blood. Interestingly, while the isolated PBMCs again showed a drastic reduction in CD16+ subsets even at the room temperature, the monocyte subset frequencies in the whole blood remained remarkably stable for up to 8 hours (**Supplementary Figure S1**). Thus, the disappearance of CD16+ monocyte subsets on culture is only seen in isolated PBMCs. As a result, factors inherent to the systemic circulation may actively preserve the CD16+ monocyte phenotype.

### Cultured CD16+ monocytes transition to the CD16-negative state without losing viability

We next asked whether CD16+ monocyte subsets disappear in isolated PBMCs because of selective cell death or loss of CD16 expression. To test this, we used FACS sorting to isolate high-purity CD16+ monocytes (**Figure 3A**) and then cultured these cells at 37 °C for 0 to 4 hours. Relative to the freshly sorted monocytes (0 hours), approximately 70% (68-77%) of the 4 hour-cultured cells demonstrated marked loss of surface CD16 expression and migrated into the flow-cytometry gate that denoted the CD14++CD16− classical monocytes (**Figure 3B**). Thus, after 4 hours of culture at 37 °C, the majority of the CD16+ cells became ‘pseudo-classical’ monocytes. Significantly, Zombie Aqua staining and flow cytometry of the sorted CD16+ monocytes showed that compared to the freshly sorted cells (0 hours), the viability of the monocytes did not change markedly over 4 hours of incubation (**Figure 3C**). Thus, the CD16+ monocytes that ‘disappear’ during standard culture are not dead; rather, they undergo a robust phenotypic transition. These data also show clearly how these cells are misclassified as classical monocytes.

**Figure 3:**
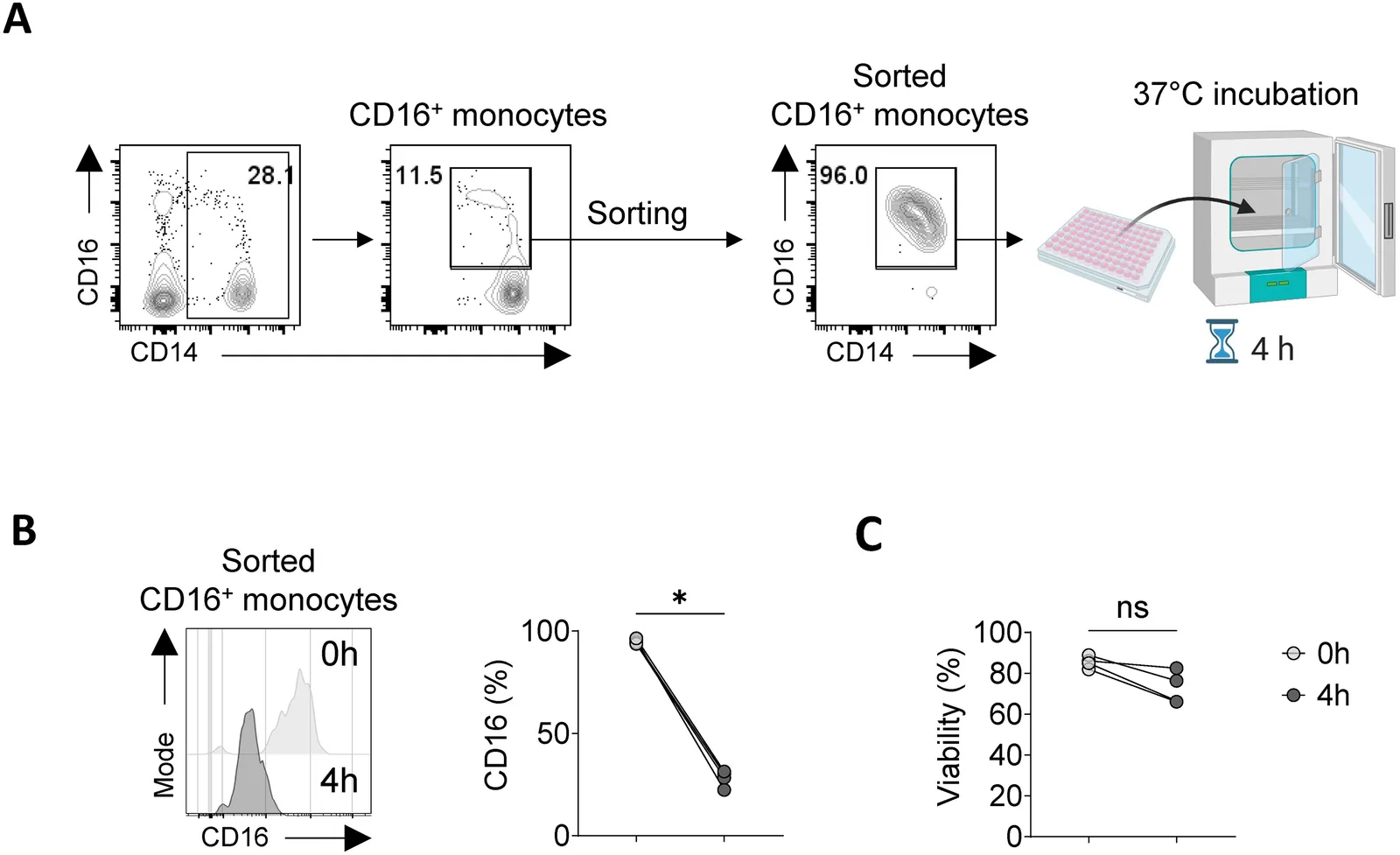
Sorted CD16+ monocytes undergo phenotypic transition to a ‘pseudo-classical’ state without loss of viability. CD16-expressing monocytes were sorted from PBMCs and cultured at 37 °C for 4 hours. **(A)** Representative flow cytometry plots and experimental scheme showing the purity of the sorted CD16+ monocytes and the incubation procedure. **(B)** Histogram of the CD16 expression on the sorted monocytes before and after 4 hours incubation (left) and plot showing the change in surface CD16 frequencies at 4 hours relative to the 0-hour timepoint (right). **(C)** Viability of the sorted CD16+ monocytes. Cells were stained with Zombie Aqua before sorting, and only Zombie Aqua-negative viable cells were collected. After 4 hours of incubation, the sorted cells were re-stained with Zombie Aqua to assess cell viability (n = 4). *p < 0.05; ns, non-significant, as determined by Mann-Whitney U test.

### Monocytes express the proteolytic machinery required for CD16 shedding

NK cells are known to shed CD16 by using the cell-membrane metalloproteinase ADAM17 (15, 16). To determine whether monocytes also possess this molecular machinery, we first asked whether monocytes can express ADAM17. For this, we analyzed the public scRNA-seq data from human PBMCs (GSE149689) (21). UMAP visualization, annotated using cell lineage markers revealed distinct immune cell clusters within PBMCs, including T cells, B cells, NK cells, and the two monocyte subsets: classical and non-classical monocytes (**Figure 4A**). When we focused on the expression of the CD16-encoding gene FCGR3A, it was predominantly localized to NK cells and the non-classical monocyte cluster (**Figure 4A**), as expected. Violin plots of the cell-type-specific expression of ADAM17 then showed that ADAM17 was predominantly expressed by the classical and non-classical monocyte cells: the lymphoid cells, including T cells, B cells, and NK cells, bore few to no ADAM17 transcripts (**Figure 4B**). To confirm this at the protein level, we assessed ADAM17 expression by intracellular flow cytometry in CD45+ cells. Monocytes accounted for approximately 75% of all ADAM17-expressing CD45+ cells, whereas CD4+ T cells, CD8+ T cells and NK cells each contributed less than 10%. Given that monocytes represent only a minor fraction of circulating CD45+ cells, this indicates a marked enrichment of ADAM17 expression in the monocyte compartment. Within the monocyte compartment, all three subsets expressed ADAM17 at substantial levels (**Figure 4C**). Thus, normal circulating monocytes are constitutively enriched in the enzymatic machinery required for CD16 cleavage.

**Figure 4:**
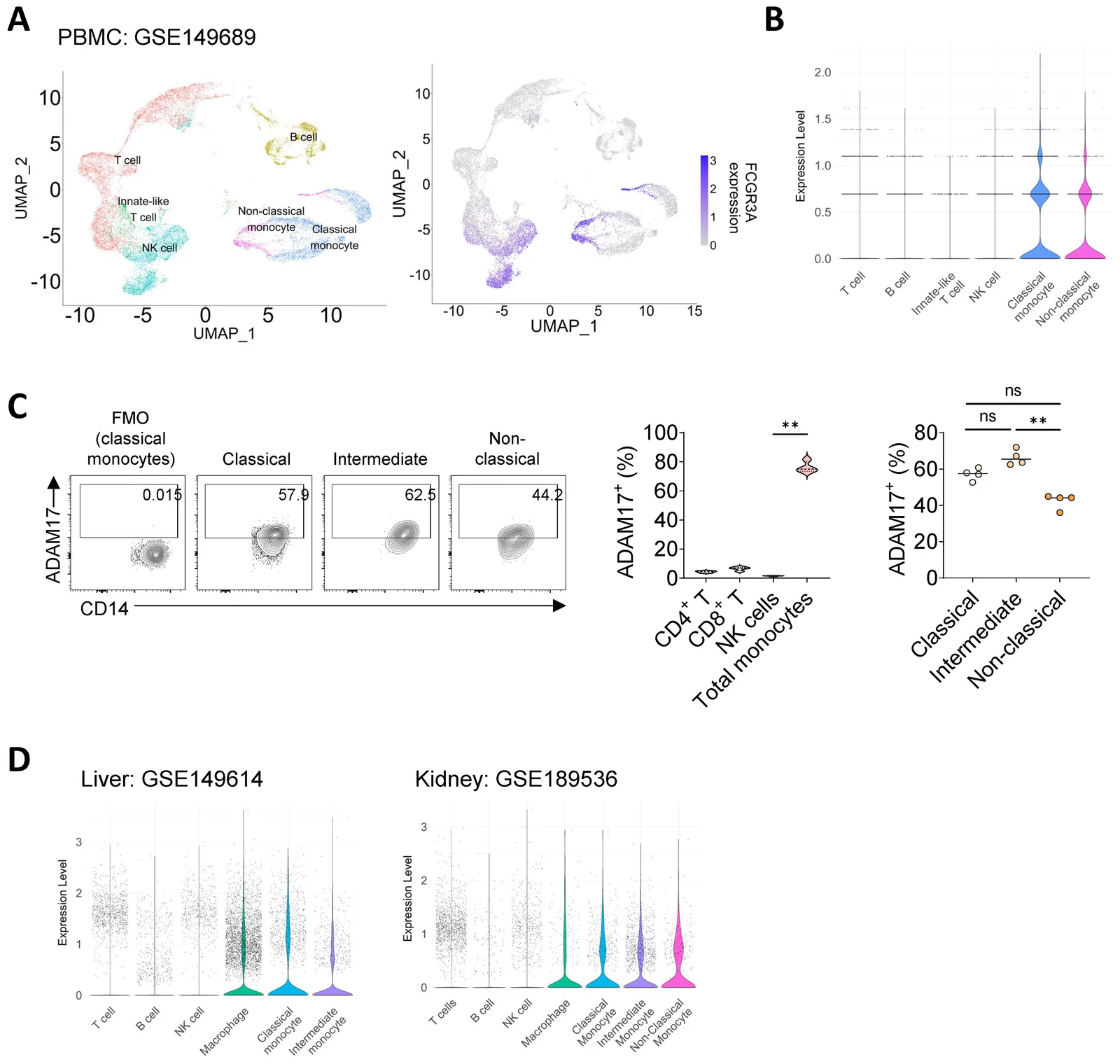
Monocytes express the proteolytic machinery required for CD16 shedding. **(A, B)** Public scRNA-seq data from human PBMCs (GSE149689) were analyzed to determine the expression of ADAM17 in the indicated immune-cell (sub)types. **(A)** UMAP clustering of PBMCs on the basis of lineage-specific marker expression, showing distinct immune cell populations including T cells, B cells, NK cells, and classical and non-classical monocytes (left), and feature plot illustrating the expression of the CD16-encoding gene *FCGR3A* in the immune-cell clusters (right). **(B)** Violin plots showing the transcript levels of *ADAM17* in the immune-cell (sub)types. **(C)** ADAM17 protein expression in human PBMCs assessed by intracellular flow cytometry. Representative flow cytometry plots of ADAM17 expression in classical, intermediate, and non-classical monocytes, with the fluorescence-minus-one (FMO) control gated on classical monocytes (left); proportions of CD4+ T cells, CD8+ T cells, NK cells, and total monocytes among ADAM17-expressing CD45+ cells (middle); and frequencies of ADAM17+ cells within each monocyte subset (right) (n = 4). **(D)** scRNA-seq data of the tissue-resident immune populations in the human liver (GSE149614) and kidney (GSE189536) were analyzed to determine the expression of *ADAM17* across immune-cell (sub)types. Violin plots illustrate the *ADAM17* transcript levels in immune-cell (sub)types in the human liver (left) and kidney (right) tissues. The cell clusters were annotated on the basis of lineage-specific markers. **p < 0.01; ns, non-significant, as determined by Kruskal-Wallis test with Dunn’s *post-hoc* correction.

Notably, our analysis of public scRNA-seq data of the tissue-resident immune populations in the human liver (GSE149614) (22) and kidney (GSE189536) (23) showed that the monocytes (and macrophages) in these tissues were also the main expressors of ADAM17 (**Figure 4D**). In the datasets examined, monocytes and macrophages were among the principal ADAM17-expressing tissue-resident immune-cell populations, although ADAM17 is also expressed by non-immune cell types, including epithelial cells (24).

### Inhibiting ADAM17 prevents loss of CD16 on monocytes in LPS-stimulated PBMCs

We next asked whether pharmacological inhibition of ADAM17 could prevent the loss of the CD16 marker on monocytes in isolated PBMCs, thereby preserving monocyte-subset frequencies. To test this, we focused on the LPS-treated PBMC model because we also wanted to assess the effect of CD16 loss during the cytokine production of monocytes by restimulation. Thus, isolated human PBMCs were treated for 5.5 hours with LPS in the presence and absence of TMI-005 or TAPI-1, which inhibit ADAM17 among other metalloproteinases (25). Flow cytometry of the fresh PBMCs (white circles in **Figure 5B**) and the PBMCs treated with LPS alone (grey circles) or with TMI-005 (red circles) showed that as expected, culture with LPS associated with the near-complete disappearance of intermediate and non-classical monocyte subsets (**Figures 5A, B**). Significantly, this loss was prevented by TMI-005 treatment (**Figures 5A, B**), and TAPI-1 showed a comparable effect (**Figure 5A**; **Supplementary Figure S2A**). Thus, TMI-005 and TAPI-1 both preserved the surface CD16 expression of LPS-treated/or cultured monocytes. These findings suggest that ADAM17 inhibition during culture can be used to maintain the original monocyte-subset identity during ex vivo experimental procedures.

**Figure 5:**
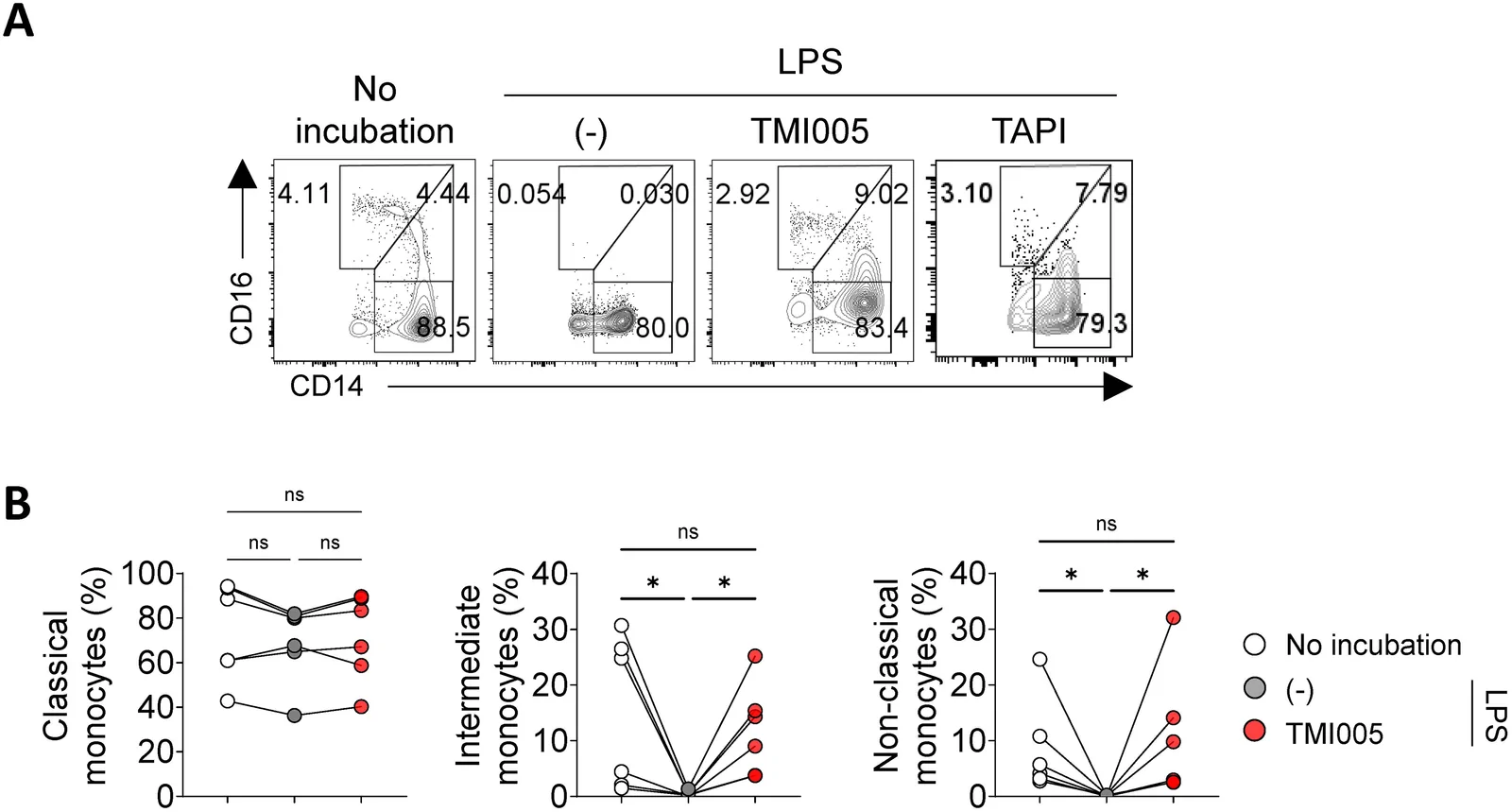
ADAM17 inhibitors preserve monocyte-subset frequencies in LPS-stimulated PBMCs. Isolated human PBMCs were stimulated with LPS (80 ng/mL) for 5.5 hours in the presence or absence of the ADAM17 inhibitor TMI-005 (1 μg/mL) or TAPI-1 (1 μg/mL). **(A)** Representative flow cytometry dot plots of the monocyte subsets in the fresh PBMCs and the PBMCs treated with LPS in the presence or absence of the indicated ADAM17 inhibitor. **(B)** Change in frequencies of classical, intermediate, and non-classical monocytes in the fresh PBMCs (white circles) and the PBMCs treated with LPS alone (grey circles) or together with TMI-005 (red circles) (n = 6). *p < 0.05, **p < 0.01; ns, non-significant, as determined by Friedman test with Dunn’s *post-hoc* correction.

To further assess the relative contributions of ADAM17 and ADAM10, we compared two structurally related hydroxamate inhibitors: GW280264X, which inhibits both ADAM10 and ADAM17, and GI254023X, which is ADAM10-preferential. Isolated PBMCs were stimulated with LPS in the presence of either compound at 1 µM. In every donor, GW280264X maintained the intermediate and non-classical subsets at approximately the frequency observed before incubation, whereas GI254023X did not significantly restore either subset (**Supplementary Figures S2B, C**). Thus, preferential inhibition of ADAM10 did not reproduce the preservation observed with combined ADAM10/ADAM17 inhibition, supporting a predominant contribution of ADAM17 to the observed CD16 loss.

### ADAM17 inhibition with TMI-005 does not broadly perturb LPS-induced cytokine responses in monocytes

Having demonstrated that both TAPI-1 and TMI-005 rescue CD16 expression via ADAM17 inhibition, we next assessed whether TMI-005 treatment itself influences monocyte function. Since TMI-005 requires dissolution in DMSO, we first determined the DMSO concentration at which LPS-induced proinflammatory cytokine production by monocytes remained unaffected. Intracellular cytokine staining revealed that 2% DMSO did not affect IL-1β or TNF-α expression; however, IL-6 expression was markedly reduced at 2% DMSO, and MCP-1 expression was significantly reduced at concentrations exceeding 1% DMSO with a non-significant reduction from as low as 0.2% DMSO (**Figure 6A**). Therefore, 0.2% DMSO was selected as the working concentration for subsequent experiments, as it did not significantly affect monocyte cytokine production.

**Figure 6:**
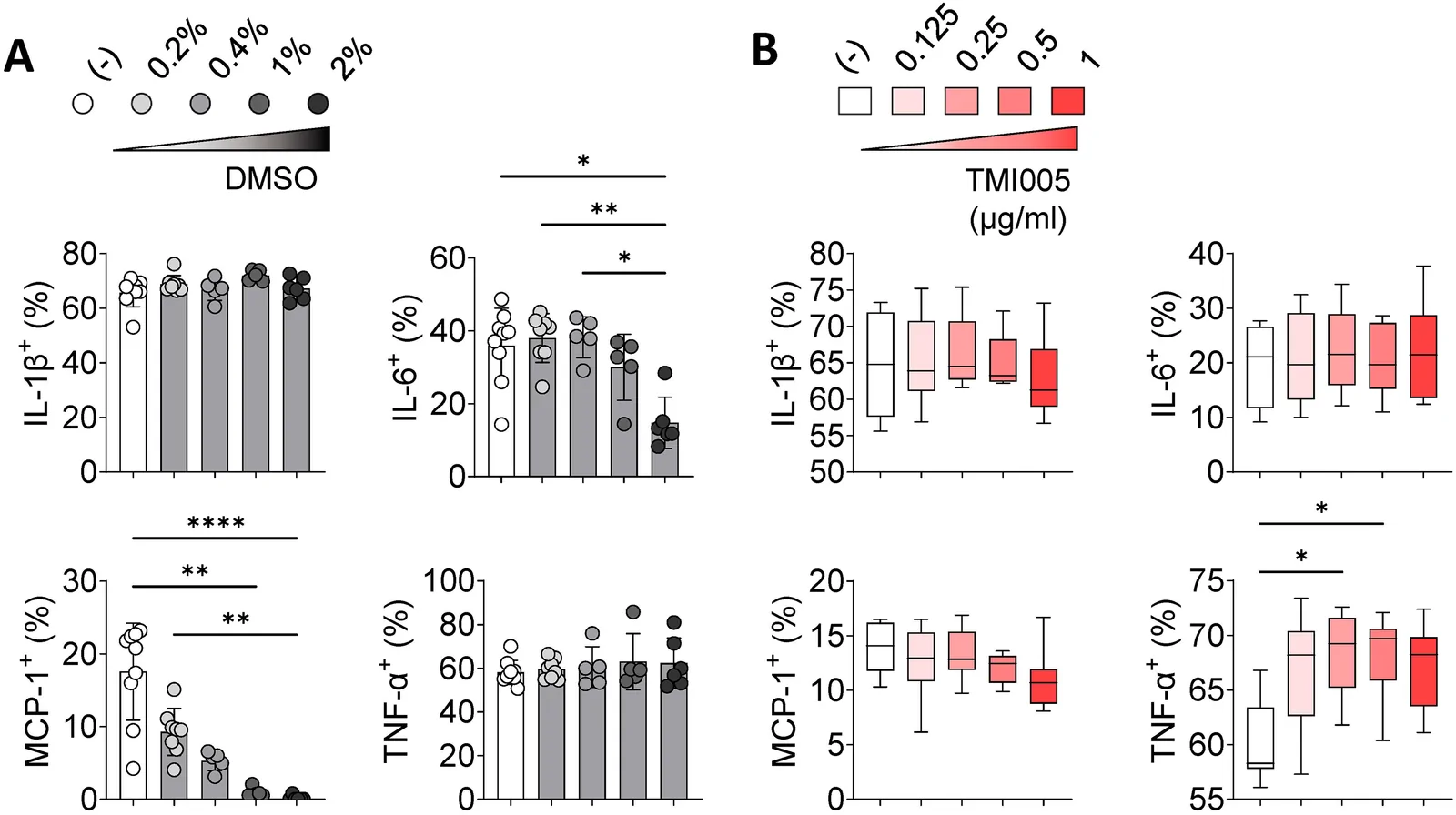
ADAM17 inhibition with TMI-005 does not impair LPS-induced cytokine production by monocytes. **(A)** PBMCs were treated with LPS (80 ng/mL) for 5.5 hours in the presence of varying concentrations of DMSO, which is the vehicle for the ADAM17 inhibitor TMI-005. The frequencies of monocytes that produced IL-1β, IL-6, MCP-1, and TNF-α were determined by flow cytometry. **(B)** PBMCs were treated for 5.5 hours with LPS (80 ng/mL) in medium containing 0.2% DMSO, or in medium containing TMI-005 (dissolved in 0.2% DMSO) at varying concentrations (0.125, 0.25, 0.5, and 1 µg/mL TMI-005). The frequencies of monocytes expressing IL-1β, IL-6, MCP-1, and TNF-α were determined by flow cytometry. The data are presented as mean ± SD (n = 5-9). *p < 0.05, **p < 0.01, ****p < 0.0001, as determined by Kruskal-Wallis test with Dunn’s *post-hoc* correction.

We next treated PBMCs with LPS in the presence of 0.2% DMSO (vehicle control) or increasing concentrations of TMI-005 (0.125, 0.25, 0.5, and 1 µg/mL; all prepared in 0.2% DMSO), and examined intracellular cytokine production. TMI-005 did not impair the capacity of monocytes to produce IL-1β, IL-6, or MCP-1 in response to LPS stimulation. Notably, TNF-α production was significantly elevated at TMI-005 concentrations of 0.25 and 0.5 µg/ml compared to the vehicle control (**Figure 6B**). Because ADAM17 is the principal sheddase for pro-TNF, this accumulation of intracellular TNF-α is most likely to reflect reduced TNF shedding rather than increased TNF production, and is consistent with effective target engagement by TMI-005 under these conditions.

This indicates that TMI-005-mediated ADAM17 inhibition at 1 µg/mL and a final DMSO concentration of 0.2% does not significantly alter the LPS-induced production of IL-1β, IL-6, or MCP-1 by monocytes, and can therefore be used to preserve monocyte subset identity during phenotypic and intracellular cytokine analyses. In addition, treatment with 1 µg/mL TMI-005 better preserved non-classical monocytes compared to other concentrations, supporting the suitability of this concentration for subsequent analyses (**Supplementary Figure S3**).

### Proposed protocol for simultaneous phenotypic and functional analysis of monocytes

Our finding that TMI-005, an ADAM17-inhibiting metalloproteinase inhibitor, blocks monocyte shedding of CD16 without significantly altering LPS-induced cytokine production suggests that the protocol proposed in **Figure 7** should be followed if researchers want to reliably determine the intracellular cytokine profiles of specific monocyte subsets. Prior to staining, isolated PBMCs were kept at 4 °C to minimize initial CD16 shedding during sample handling. (i) CD16 surface staining was performed together with LPS stimulation and TMI-005 treatment. Although surface staining can be performed prior to LPS and TMI-005 treatment, simultaneous treatment is recommended for improved preservation of intermediate and non-classical monocytes (**Supplementary Figures S4A, B**). (ii) No additional TMI-005 was required following fixation and permeabilization. (iii) Intracellular cytokine staining was subsequently performed, and classical, intermediate, and non-classical monocyte populations were analyzed by flow cytometry. Since TMI-005 must be dissolved in DMSO, it should be used as a concentration of 1 µg/mL, which equates to 0.2% DMSO. Negative controls should therefore consist of medium with 0.2% DMSO. We believe this protocol will help researchers to overcome the long-standing problem of monocyte-subset misclassification in clinical and basic immunology studies.

**Figure 7:**
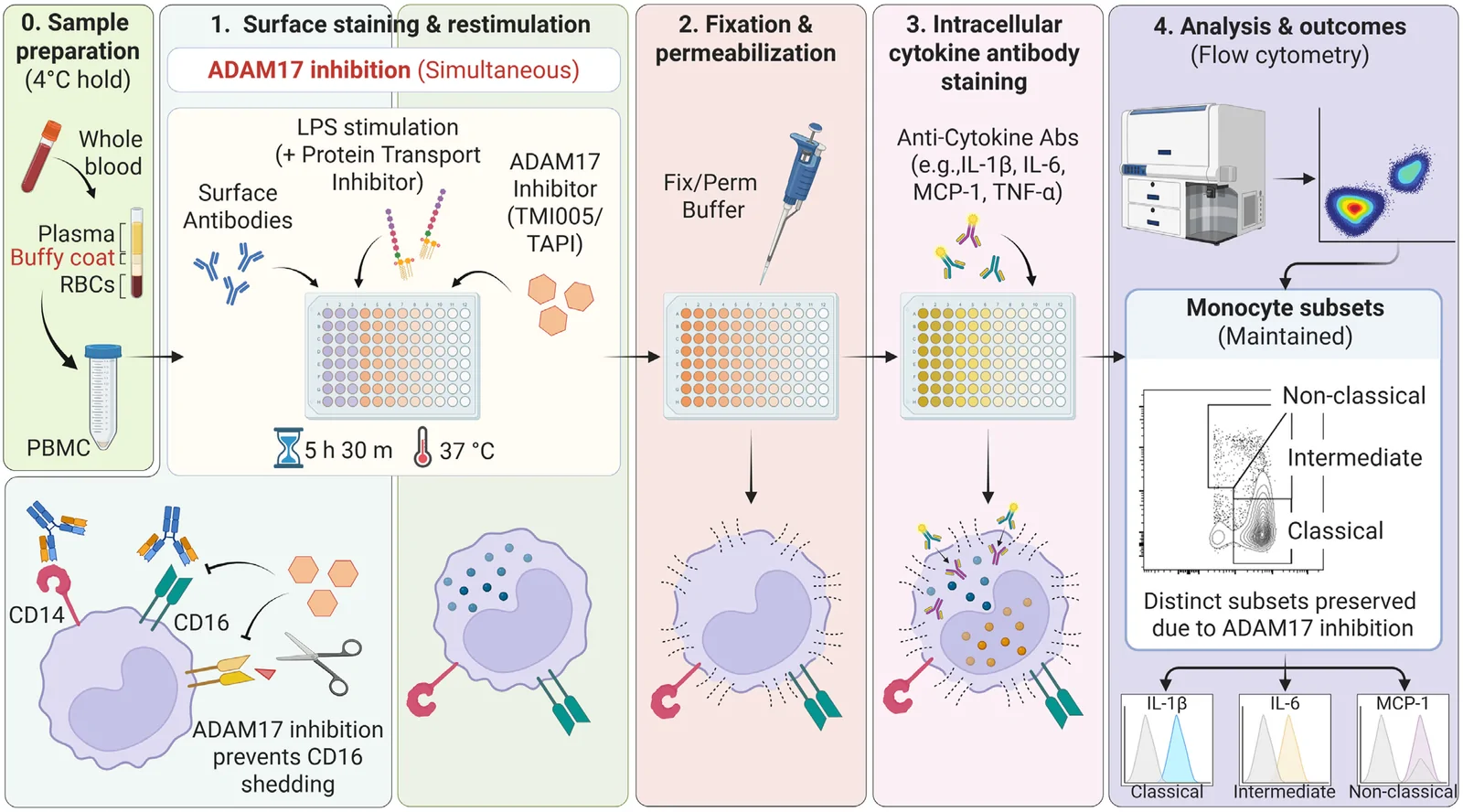
Proposed experimental protocol for functional characterization of human monocyte subsets. Schematic depiction of the optimized procedure for preserving CD16-defined monocyte subset identity during intracellular cytokine analysis. The protocol consists of the following steps: (0) Sample preparation: PBMCs are either freshly isolated or thawed from cryopreserved stocks, and are maintained at 4 °C until use, to minimize CD16 shedding during sample handling. (1) Surface antibody staining, LPS stimulation (80 ng/mL), ADAM17 inhibitor treatment (TMI-005, 1 μg/mL), and a protein transport inhibitor (monensin) are applied simultaneously at 37 °C for 5.5 hours. (2) Cells are fixed and permeabilized using a fixation/permeabilization buffer at 4 °C. (3) Intracellular cytokine staining is performed. (4) Analysis: classical, intermediate, and non-classical monocytes are resolved by flow cytometry and their cytokine production is quantified.

## Discussion

The three human monocyte subsets have distinct functional roles and are conventionally distinguished by their surface expression of CD14 and CD16 (1, 2). However, we showed that CD16 expression disappears when ex vivo intermediate and non-classical monocytes are exposed to proinflammatory stimuli or just simply cultured. The shift of these cells to a ‘pseudo-classical’ phenotype occurs rapidly: most of the intermediate and non-classical monocytes apparently ‘disappear’ from the cultured cell population within 4 hours. These findings confirm previous studies (17, 18) and lead to questions about the reliability of CD16 as a lineage marker of monocyte subsets.

While Waller et al. first reported that ADAM17 inhibition with TMI-005 could reverse the apparent loss of CD16+ intermediate monocytes following TLR agonist stimulation in the context of acute inflammatory disease (18), our study substantially extends these findings in several important ways. First, we demonstrate that CD16 shedding occurs not only under inflammatory stimulation but also during simple ex vivo culture in the absence of any stimulus, and that this process is strictly temperature-dependent, occurring within 1–4 hours at 37 °C but not at 4 °C. Second, pre- versus post-incubation labeling experiments argue against receptor internalization as the major explanation for the observed CD16 loss, supporting proteolytic cleavage as a key mechanism. Third, we reveal that monocytes in unseparated whole blood maintain stable CD16 expression for up to 8 hours, implicating plasma-derived factors such as TIMP-3 as endogenous regulators of ADAM17 activity in the circulation. Fourth, we extend the characterization of ADAM17 expression beyond peripheral blood monocytes to tissue-resident immune populations in the liver and kidney using publicly available scRNA-seq datasets, supporting a broader role for monocyte-intrinsic ADAM17 across tissues. Finally, and most critically, we translate these mechanistic insights into a validated, step-by-step experimental protocol that integrates ADAM17 inhibition into simultaneous surface staining, LPS stimulation, and intracellular cytokine profiling — a practical tool that has not previously been established for human monocyte subset analysis. Moreover, the ADAM10-preferential inhibitor GI254023X did not restore the CD16+ subsets, which further supports ADAM17 as the main enzyme responsible for CD16 loss, although a contribution of ADAM10 cannot be completely excluded.

Thus, ex vivo CD16+ monocyte ‘disappearance’ is due to swift enzyme-mediated proteolysis that converts these cells into a ‘pseudo-classical’ phenotype. This mechanism was supported by our in silico analyses of publicly available scRNA-seq data on human PBMCs: these analyses showed that of all immune cells in the peripheral blood, monocytes constitutively express the highest levels of ADAM17. Our flow cytometric analysis confirmed this at the protein level: monocytes accounted for approximately three-quarters of ADAM17-expressing CD45+ cells, and all three monocyte subsets expressed ADAM17. Thus, CD16 loss in each subset may depend not only on ADAM17 expression but also on the amount of CD16 available for cleavage, which is low in classical monocytes. These findings led us to develop a CD16 stabilization protocol for ex vivo monocyte analyses, where all ex vivo manipulations are conducted in the presence of TMI-005, an ADAM17 inhibitor. This protocol effectively blocks CD16 shedding, thus eliminating a critical methodological limitation that confounds monocyte research.

Our study confirms previous reports suggesting that monocytes can lose CD16 during ex vivo handling and proinflammatory stimulation (17, 18, 26). These findings suggest that the significant changes in non-classical monocyte frequencies observed in various inflammatory conditions (8) may actually reflect artifactual ‘pseudo-classical’ shifting rather than genuine in vivo phenotypic changes. These findings have significant implications for the interpretation of existing literature. Numerous clinical studies have reported reductions in CD16+ monocyte subsets in conditions such as acute pancreatitis (18), acute myocardial infarction (27), and viral infections including COVID-19 (28), and have attributed these changes to genuine in vivo immunological shifts. In light of our findings, however, it is plausible that at least a proportion of these reported changes reflect ex vivo CD16 shedding induced during sample processing rather than true alterations in monocyte subset composition. This methodological artifact is particularly insidious because it is invisible without appropriate controls: cells that have undergone ADAM17-mediated CD16 cleavage are indistinguishable from genuine classical monocytes by standard CD14/CD16 gating. The ADAM17 inhibitor-based protocol proposed here provides a straightforward and cost-effective means to resolve this ambiguity, and we strongly advocate for its adoption as a useful standardization strategy for ex vivo studies relying on CD14/CD16-based monocyte subset analysis.

NK cells also demonstrate ADAM17-mediated cleavage. It is well documented that this regulatory mechanism occurs rapidly after NK cell activation. It helps NK cells detach from their target cells, thus preventing NK cell overstimulation (15, 29). It is possible that CD16 shedding by monocytes also plays important physiological roles: for example, it could help modulate the environmental sensing of monocytes or prevent their over-activation during inflammation. Further studies are needed to elucidate these putative regulatory mechanisms. In addition, ADAM17 cleaves other substrates, including pro-TNF, CD62L, and the TNF receptors (30). Indeed, intracellular TNF-α accumulated in TMI-005-treated monocytes (**Figure 6B**), which likely reflects impaired processing of pro-TNF. Therefore, the proposed protocol should be used with caution in assays that assess adhesion, migration, or TNF responsiveness, or CD16-dependent functions such as antibody-dependent cellular phagocytosis, as these were not examined in this study.

One striking observation in our study was that monocytes shed CD16 during simple incubation if they were in the context of PBMCs but not if they were in the context of whole blood. Indeed, CD16 expression on monocyte subsets in blood remained remarkably stable for up to 8 hours. Since circulating monocytes retain CD16 at 37 °C, the temperature dependence observed in isolated PBMCs may reflect the loss of an in vivo mechanism that restrains ADAM17 activity, rather than an absence of enzyme activity at body temperature. This indicates that whole blood bears factors that prevent CD16 shedding. One possibility is that human plasma contains Tissue Inhibitor of Metalloproteinase-3 (TIMP-3) (31), which is a potent endogenous inhibitor of ADAM17. TIMP-3 regulates its target metalloproteinases by facilitating their sequestration in cells via endocytic receptors such as LRP-1 (32). Thus, whole blood may buffer ADAM17 activity on monocytes. This suggests that for basic or clinical monocyte research, it is preferable to leave monocytes/PBMCs in whole blood and only isolate them immediately before flow cytometry. However, we also showed that storing PBMCs at 4 °C markedly prevented CD16 loss on monocytes. Thus, we propose that if monocytes/PBMCs cannot be isolated from whole blood just before experimental use, they should be immediately placed at 4 °C after isolation. Monocyte subset frequencies remained stable for at least 12 hours in PBMCs at 4 °C and for at least 8 hours in whole blood at room temperature, and longer holding times should be validated by each laboratory. In addition, Johnson et al. reported that monocyte subsets are largely preserved in whole blood kept at ambient temperature for 24 hours (26), which is relevant to overnight shipping. However, the effect of cryopreservation for longer than four weeks remains to be determined.

Because CD16 must be labeled during stimulation to preserve subset identity, we cannot exclude the possibility that binding of the anti-CD16 antibody induces FcRγ-dependent signaling, although such signaling generally requires cross-linking and differs between antibody clones (33). Thus, a control without the CD16 antibody may be needed when other clones or stimulation conditions are used. Nevertheless, this approach is preferable to staining after stimulation, which causes intermediate and non-classical monocytes to be misclassified as classical monocytes.

Our findings relate to the stability of surface CD16, not to the developmental relationship between monocyte subsets. In vivo, classical monocytes mature into intermediate and then non-classical monocytes over several days (9, 10), whereas the CD16 loss described here occurs within hours of ex vivo handling. Therefore, changes in subset frequencies after sample handling should not be interpreted as changes in the cells themselves without additional evidence. Accordingly, our data do not argue for replacing the widely used CD14/CD16 classification, but rather define the handling conditions under which it can be reliably applied.

Given the instability of CD16, several studies have sought to identify marker combinations that will allow accurate classification of monocyte subsets. For instance, Ong et al. suggest to use CD33, CD86, CD64, HLA-DR, and CCR2 (17). Specifically, CD33, CD86, and CD64 are used to enrich for monocytes and exclude non-monocytes; CD33 expression also helps separate non-classical from intermediate monocytes; CD64 gating also removes DC contamination; CCR2^high^ identifies classical monocytes; and HLA-DR^high^ in the CD64^high^ compartment indicates intermediate monocytes (17). Moreover, 6-sulfo LacNAc (slan) expression has been proposed as a potential marker to distinguish non-classical monocytes (which express slan) from intermediate monocytes (which do not) (34, 35). However, these classification approaches involve more complex flow cytometric strategies and the CD16-based classification system continues to be widely used (1). Thus, our ADAM17 inhibitor-based stabilization protocol is a simple and cost-effective strategy for preserving the ‘true’ *in vivo* phenotype.

In conclusion, we showed that intermediate and non-classical monocytes in isolated PBMCs transition rapidly into a ‘pseudo-classical’ phenotype due to ADAM17-mediated shedding. This affects blood monocytes, possibly because PBMC isolation immediately removes circulating ADAM17 inhibitors such as TIMP-3. The loss of CD16 during monocyte/PBMC culture is a serious methodological limitation that may confound the interpretation of studies that rely on CD16-based subset identification after *ex vivo* manipulation. We propose that ADAM17 inhibition should be considered as part of early-stage processing protocols when CD16-defined monocyte subsets are analyzed after *ex vivo* manipulation.

## Data Availability

The raw data supporting the conclusions of this article will be made available by the authors, without undue reservation.

## Supplementary material

Supplementary Figure 1 Monocytes in isolated PBMCs, but not in whole blood, lose CD16 expression at room temperature. Human blood was either kept as unseparated whole blood or processed to isolate PBMCs. Isolated PBMCs were resuspended in complete RPMI 1640 medium and both samples were incubated at room temperature and analyzed at 0, 1, 2, 4, and 8 hours. **(A)** Representative flow-cytometry plots of the monocyte subsets. **(B)** Frequencies of the classical, intermediate, and non-classical monocyte subsets from a representative experiment.

Supplementary Figure 2 Comparison of metalloproteinase inhibitors for the preservation of CD16-expressing monocyte subsets. **(A)** Isolated human PBMCs were stimulated with LPS (80 ng/mL) for 5.5 hours in the presence of TMI-005 (1 μg/mL) or TAPI-1 (1 μg/mL). The changes in intermediate (ΔIM) and non-classical (ΔNCM) monocyte frequencies induced by each inhibitor relative to the LPS-only condition (Drug − LPS) are shown (n = 3). **(B, C)** Isolated human PBMCs were stimulated with LPS (80 ng/mL) for 5.5 hours in the presence or absence of the ADAM10/ADAM17 inhibitor GW280264X (1 μM) or the ADAM10-preferential inhibitor GI254023X (1 μM). **(B)** Representative flow cytometry dot plots of the monocyte subsets in the fresh PBMCs (No incubation) and the PBMCs treated with LPS in the presence or absence of the indicated inhibitor. **(C)** Frequencies of classical, intermediate, and non-classical monocytes (n = 6). *p < 0.05, **p < 0.01; ns, non-significant, as determined by Mann-Whitney U test **(A)** or Friedman test with Dunn’s *post-hoc* correction **(C)**.

Supplementary Figure 3 TMI-005 at 1 µg/mL optimally preserves monocyte subset distribution across tested concentrations. The ability of TMI-005 to preserve monocyte subset distribution was evaluated across different concentrations to determine the optimal working concentration. PBMCs were treated with TMI-005 at the indicated concentrations (0.125, 0.25, 0.5, and 1 µg/mL) in medium containing 0.2% DMSO and stimulated with LPS (80 ng/mL) at 37 °C in the presence of surface antibodies for 5.5 hours. Frequencies of classical, intermediate, and non-classical monocyte subsets are shown. The data are presented as mean ± SD (n = 8). Statistical significance was determined by the Kruskal-Wallis test with Dunn’s *post-hoc* correction.

Supplementary Figure 4 Comparison of pre- vs co-surface staining in TMI-005 and LPS stimulation. Frequencies of intermediate and non-classical monocytes were compared under pre- and co-surface staining conditions. Fresh PBMCs without any incubation were included as no-incubation control. For pre-staining, PBMCs were first incubated with surface antibodies for 20 minutes, followed by stimulation with LPS (80 ng/mL) at 37 °C in the presence of either 0.2% DMSO or TMI-005 (1 µg/mL in 0.2% DMSO) for 4 hours. For co-staining, PBMCs were stimulated with LPS (80 ng/mL) at 37 °C for 4 hours in the presence of surface antibodies together with either 0.2% DMSO or TMI-005 (1 µg/mL in 0.2% DMSO). **(A)** Representative dot plots. **(B)** Quantification of classical, intermediate and non-classical monocyte frequencies. The data are presented as mean ± SD (n = 5). *p < 0.05, as determined by Kruskal-Wallis test with Dunn’s *post-hoc* correction.
